# A novel dual-task paradigm with story recall shows significant differences in the gait kinematics in older adults with cognitive impairment: A cross-sectional study

**DOI:** 10.3389/fnagi.2022.992873

**Published:** 2022-12-15

**Authors:** Nawab Ali, Jin Liu, Huifang Tian, Wei Pan, Yao Tang, Qian Zhong, Yaxin Gao, Ming Xiao, Han Wu, Cuiyun Sun, Ting Wu, Xi Yang, Tong Wang, Yi Zhu

**Affiliations:** ^1^Rehabilitation Medicine Center, The First Affiliated Hospital of Nanjing Medical University, Nanjing, China; ^2^Clinical Medicine Research Institution, The First Affiliated Hospital of Nanjing Medical University, Jiangsu Province Hospital, Nanjing, China; ^3^School of Rehabilitation Medicine, Nanjing Medical University, Nanjing, China; ^4^Rehabilitation Department, Daishan Community Health Service Center, Nanjing, China; ^5^Rehabilitation Medicine Department, Geriatric Hospital of Nanjing Medical University, Nanjing, China; ^6^Department of Rehabilitation, Nanjing Drum Tower Hospital Clinical College of Nanjing Medical University, Nanjing, China; ^7^Department of Rehabilitation, Suzhou Municipal Hospital, Gusu School, The Affiliated Suzhou Hospital of Nanjing Medical University, Nanjing Medical University, Suzhou, China; ^8^Jiangsu Key Laboratory of Neurodegeneration, Center for Global Health, Nanjing Medical University, Nanjing, China; ^9^Brain Institute, The Affiliated Nanjing Brain Hospital of Nanjing Medical University, Nanjing, China; ^10^Center of Global Health, Nanjing Medical University, Nanjing, China; ^11^Neurology Department, The First Affiliated Hospital of Nanjing Medical University, Nanjing, China

**Keywords:** mild cognitive impairment, subjective cognitive decline, motor dysfunctions, dual-tasking, kinematics, gait

## Abstract

**Objective:**

Cognitive and motor dysfunctions in older people become more evident while dual-tasking. Several dual-task paradigms have been used to identify older individuals at the risk of developing Alzheimer’s disease and dementia. This study evaluated gait kinematic parameters for dual-task (DT) conditions in older adults with mild cognitive impairment (MCI), subjective cognitive decline (SCD), and normal cognition (NC).

**Method:**

This is a cross-sectional, clinical-based study carried out at the Zhongshan Rehabilitation Branch of First Affiliated Hospital of Nanjing Medical University, China.

**Participants:**

We recruited 83 community-dwelling participants and sorted them into MCI (*n* = 24), SCD (*n* = 33), and NC (*n* = 26) groups based on neuropsychological tests. Their mean age was 72.0 (5.55) years, and male–female ratio was 42/41 (*p* = 0.112). Each participant performed one single-task walk and four DT walks: DT calculation with subtracting serial sevens; DT naming animals; DT story recall; and DT words recall.

**Outcome and measures:**

Kinematic gait parameters of speed, knee peak extension angle, and dual-task cost (DTC) were obtained using the Vicon Nexus motion capture system and calculated by Visual 3D software. A mixed-effect linear regression model was used to analyze the data.

**Results:**

The difference in gait speed under DT story recall and DT calculation was −0.099 m/s and − 0.119 m/s (*p* = 0.04, *p* = 0.013) between MCI and SCD, respectively. Knee peak extension angle under DT story recall, words recall, and single task was bigger in the MCI group compared to the NC group, respectively (*p* = 0.001, *p* = 0.001, *p* = 0.004). DTC was higher in the DT story recall test than all other DT conditions (*p* < 0.001).

**Conclusion:**

Kinematic gait parameters of knee peak extension angle for the DT story recall were found to be sensitive enough to discriminate MCI individuals from NC group. DTC under DT story recall was higher than the other DT conditions.

## Introduction

Aging is associated with an increased risk of physical and cognitive decline, which can lead to cognitive and motor dysfunction (Anton et al., 2015, p. 58). By 2050, the number of people of aged 65 and older with Alzheimer’s disease and dementia that will significantly contribute to disability and loss of independence is projected to reach 12.7 million worldwide (Wollesen et al., 2019). The human gait pattern is affected by age and cognitive decline. For example, older individuals walk slowly, have a shorter step and stride length, wider steps, and high gait variability (Li and Lindenberger, 2002; Herssens et al., 2018). In older people, safe walking and maintaining a proper speed require intact cognition and executive control and is an indicator of general health and survival. This is because the sensorimotor aspect of walking requires a high degree of attention and cognitive control (Cullen et al., 2019). A growing body of evidence suggests that gait impairment is clinically significant and can predict cognitive decline earlier than cognitive tests (Montero-odasso et al., 2005; Porta et al., 2020).

Understanding the relationship between gait and cognitive impairment has broad public health implications for the aging population (Al-Yahya et al., 2011). The activities of daily life usually involve simultaneous cognitive and motor performance or dual-tasking. Such activities like walking while talking and avoiding obstacles or making turns, become challenging with advancing age (Mancioppi et al., 2020). Dual-task performance can predict the deterioration of gait and cognitive decline in people with neurological deficits. Studies have shown that the slowing of gait during dual-tasking can differentiate healthy individuals from people with neurological problems such as pre-dementia or mild cognitive impairment (MCI; Dubost et al., 2006). Poor dual-task (DT) performance was also found to be associated with an unstable gait and a high risk of fall in the frail elderly, and has been considered a predictor of future fall (Fuentes-Abolafio et al., 2020). Recently, it has been found that motor impairments precede cognitive impairment and that early motor changes such as gait speed and dual-task cost (the percentage difference between single and dual-task performance in cognitive and/or motor tasks) are potential biomarkers for the progression of cognitive decline from MCI to Alzheimer’s disease (Montero-Odasso and Perry, 2019; Bishnoi and Hernandez, 2021). With advancing age and deteriorating physical functions, older adults become heavily dependent on cognitive reserve (Bishnoi and Hernandez, 2021). Many studies suggest that increased cognitive demand under DT conditions increases the sensitivity of gait assessment (Ramírez and Gutiérrez, 2021). Thus, gait dysfunction in combination with memory, execution, and attention-demanding tasks may be used to predict and distinguish individuals with pathological cognitive decline from healthy individuals. Several DT paradigms, such as walking and simultaneously performing arithmetic (counting, subtracting), verbal (calling animal names), and memory (words recall) tasks, have been used to investigate the interaction between gait and cognition (Montero-Odasso et al., 2017; Åhman et al., 2020). A recent systemic review showed the mental tracking tasks including serial subtraction and verbal fluency were the most sensitive in detecting MCI-related changes in older adults (Bishnoi and Hernandez, 2021). Although the “words per time unit” outcomes of DT tests including Timed-Up-and-Go (TUGdt), i.e., “animals/10 s” and “months/10 s” were found to have high levels of discrimination between dementia, MCI, subjective cognitive decline (SCD), and normal cognition (NC) groups, the DTC showed no difference among groups (Åhman et al., 2020). Another study found that DT parameters under words recall cannot distinguish MCI from normal elderly either (Jayakody et al., 2020).

Studies indicated that functional changes in gait can be easily identified through kinematic analysis (Muir et al., 2012; Beauchet et al., 2016). They have found that gait kinematics of the lower limb changes with cognitive decline and become worse with the progression of the disease. Another study has found that gait speed was associated with immediate recall memory in older adults (Sebastiani et al., 2020). Spatiotemporal gait variations using the DT paradigm are well studied in MCI patients, and most studies have reported changes in gait speed under those conditions (Mintun et al., 2021), (Montero-Odasso et al., 2020). Fuentes-Abolafio et al. (2021) have reported that MCI patients have higher variability in kinematic parameters compared to healthy adults. However, further studies are needed to find which kinematic parameters are sensitive enough to discriminate people with MCI from healthy individuals. Furthermore, changes in joint kinematics between single and dual-tasking have not been reported, and such observations could be relevant for targeting specific interventions for the prevention of functional and cognitive decline. Since memory is typically impaired in people with MCI, dual-tasking involving memory tasks may help to distinguish MCI patients from healthy individuals. In our previous study, story recall has a higher DTC compared to words recall in MCI and normal cognitive elderly, and DTC of words recall in MCI group was significantly higher than it in the NC group (Zhu et al., 2020). The difference of DTC under DT story recall failed to reach a significance level, which may be due to a small sample size. We therefore hypothesize that (1) a novel dual-tasking with story recall can distinguish MCI patients from healthy individuals better than the other DTs including calculation, naming animals, and words recall, and (2) joint kinematic parameters under a DT conditions are different in MCI and SCD patients compared to healthy older adults. The aims of this study were to identify the significance of DT paradigm with story recall in older adults and to assess whether kinematic gait parameters such as gait speed, knee peak extension angle, and DTC can differentiate patients with MCI from SCD and cognitively normal older adults.

## Methodology

### Participants selection criteria

Older adults from the local community were recruited if they: (1) were 55–85 years old; (2) had no neurological disease such as stroke, severe head injury, or cerebral tumor; (3) had no lower limb functional mobility issues, fractures, diabetic foot, or severe arthritis; (4) had no severe cardiopulmonary problems; (5) had no serious liver or kidney dysfunction; and (6) had received primary education or above. Participants were excluded if they had any of the following conditions: (1) had structural abnormalities such as brain tumor, subdural hematoma, head trauma, or a neurological or psychiatric disorder that could impact cognitive functions; (2) had severe depression or were unable to participate in cognitive function tests or gait analysis; or (3) had communication problems such as deafness, blindness, or language problems.

### Screening and recruitment

#### Sample size calculation

The sample size was calculated using PASS 15 with repeated measures analysis procedure. The outcome was DTC. The mean DTC of DT calculation, DT naming animals, DT story memory, and DT words memory were 0.14, 0.14, 0.19, and 0.12 which were between subject effect, the mean DTC of MCI, SCD, and NC group were 0.15, 0.1, and 0.1 which was within-subject effect. The standard deviation of effects was set 0.02, the between-subject standard deviation was 0.1 and the auto correlation was 0.2. To achieve 80% power at a 2-sided significance level of 5%, the sample size of each group was 25, and the overall sample size was 75. Considering 5% of withdraw from the study, the sample size of each group was 26, and the overall sample size was 78.

Participants were screened by a neuropsychologist from July 2020 to June 2021 at the memory clinic of the First Affiliated Hospital of Nanjing Medical University. For this cross-sectional study, the screened individuals were recruited if they met the diagnostic criteria for MCI, SCD, or NC and provided written consent. The Mini-Mental State Examination (MMSE) and Clinical Dementia Rating (CDR) scores were used to exclude dementia and Alzheimer’s disease patients (Folstein et al., 1975; Morris, 1993; Lam et al., 2008). The Hachinski ischemic score (HIS) was also administered to exclude vascular mild cognitive impairment or dementia (Hachinski et al., 2012).

The cognitive status of the participants was assessed on three cognitive domains: (1) memory (delayed recall and delayed recognition score based on the Huashan version of the auditory-verbal learning test, AVLT-H; Zhao et al., 2012); (2) speed/executive function (time spent on Trial Making Tests, TMT-A, and TMT-B; Salthouse, 2011); and (3) language function (verbal fluency test and Boston Naming Test, BNT; Stålhammar et al., 2015). Furthermore, depression was assessed using the Chinese version of the Geriatric Depression Scale (GDS-30; Chau et al., 2006).

The diagnostic criterion for MCI was based on the above neuropsychological tests (Bondi et al., 2014), recommendations for diagnosis and treatment of preclinical Alzheimer’s disease in China, and having memory complaints for more than 6 months (Han, 2018). In addition, a self-reported questionnaire was used to distinguish SCD from NC individuals according to the suggestions of the SCD-Initiative working group.

Participants were considered to have MCI if they had at least one of the following: (1) two impaired scores on any two scales of the three cognitive domains (memory, speed/executive function, or language of >1 SD below the age-corrected normative means) or (2) one impaired score in each of the three scales of cognitive domains (memory, speed/executive function, or language, >1 SD below the age-corrected normative mean in each of the three cognitive domains). The normative means selected in this study are taken from Chinese population studies as described by Li et al. (2019).

Individuals were considered to be SCD if they met the following criteria (Slot et al., 2018; Cullen et al., 2019): (1) had a self-reported persistent decline in the memory domain of cognition for more than 6 months; (2) had concerns about memory loss and feeling of deteriorating performance compared to individuals of the same age group; (3) had worse performance on standard cognitive tests adjusted for age, gender, and education; and (4) did not meet MCI or dementia diagnostic criteria.

The inclusion criteria for healthy individuals (NC) were as follows: (1) they had no complaints of cognitive impairment or memory loss, and (2) they did not meet SCD or MCI diagnostic criteria.

This study was approved by the Ethics Committee of the First Affiliated Hospital of Nanjing Medical University (also named Jiangsu Province Hospital; Approval Number: 2019-SR-015). All of the participants provided written consent.

### Motion capture and gait assessment of the ST and DT walking

All participants completed one ST and four DT walking tasks. For the ST, participants were asked to walk at their usual pace in a quiet, well-lit room wearing comfortable footwear and without the use of any mobility aids. For the DTs, participants walked at their usual pace while also performing the following cognitive tasks aloud: DT calculation, DT naming animals, DT story recall, and DT words recall. In DT calculation, participants were required to count down from 100, 90, 80, and 70 by serial 7 s while walking. In DT naming animals, participants were asked to say out loud as many names of animals as possible while walking. In DT story recall, participants were required to repeat a short story while walking, narrated to them at the beginning of the test. In DT words recall, participants were asked to repeat five Chinese words (narrated at the beginning of the test) during walking. These four DT paradigms were repeated three times for each participant to obtain at least ten gait cycles of data for each participant.

Gait observation of the participants was carried out at the Gait Lab in the Zhongshan Rehabilitation Branch of First Affiliated Hospital of Nanjing Medical University. A Vicon Nexus 2.8 (with 12 cameras, Vantage5, Vicon Nexus2.8, Oxford Metrics, Oxford, United Kingdom) motion capture system was used to collect movement data. The Conventional Gait Model 2 (CGM 2.3 vision), an open-source biomechanical model with 51 markers, was used to capture the gait data. These markers were attached to different parts of the body, the details of which have been previously published (Zhong et al., 2021). Participants were instructed to walk at their usual speed on a 10 m walking path. To reduce the impact of acceleration/deceleration and turning on walking speed, the 2-m window at the beginning and end of the walking test was not included in the final data collection. To minimize the effects of fatigue, participants were allowed 2–3 min rest between the tasks. Time taken by the subjects during the middle 6 m window was noted and retained by the motion capture system to obtain gait kinematics for further analysis.

### Kinematic analyses of gait data

Gait kinematic parameters and average speed were processed using Visual 3D software (C-motion Inc., Rockville, MD, United States), and kinematic variables were recorded for right and left legs separately. We further used the captured motion to define heel contact and toe-off for stride and step identification, as well as joint angle identification between the shank and thigh in the sagittal plane. We also used it to calculate the average level waking speed, knee peak extension angle, and DT cost. Dual-task cost (DTC) was obtained using gait speed for each individual in all dual-task conditions. DTC is the measure of reduced walking performance (slowing of gait speed) due to cognitive-motor interference while dual-tasking. It is the percentage of decrement in performance between ST and DTs. DTC was calculated using the gait speed under ST and DT with the following formula: DTC = [(ST gait speed – DT gait speed)/ST gait speed] (Cullen et al., 2019).

### Statistical analysis

The demographic characteristics of the participants are described in Table 1. Categorical variables are presented as proportions and were compared using the *χ*^2^ test. Continuous variables are shown as the mean, median and interquartile range, as well as standard deviation and confidence interval (minimumand maximum), and their distribution was examined using the Wilcoxon rank-sum test. We used a linear mixed, random-effects model, a random slope (for different tasks), and unstructured correlation to estimate change in gait parameters under different tasks and cognitive status. Gait parameters, i.e., gait speed, knee peak extension angle, and DTC were considered as dependent variables, while various tasks and cognitive status as independent variables. We had pre-selected, gender, age, body mass index (BMI), diabetes, GDS score, and years of education as potential covariates, based on the literature review and our previous findings. A two-sided *p* < 0.05 was considered statistically significant. All statistical analyses were performed using the statistical software SAS 9.4.

**Table 1:** Baseline characteristics of the participants.

Variables	MCI (*N* = 24)	SCD (*N* = 33)	NC (*N* = 26)	Total (*N* = 83)	Value of *p*
Sex, *n* (%)					
Male	12 (50.0)	15 (45.5)	15 (57.7)	42 (50.6)	0.112
Female	12 (50.0)	18 (54.5)	11 (42.3)	41 (49.4)	
Age, years					
N (Nmiss)	24 (0)	33 (0)	26 (0)	83 (0)	
Mean (STD)	71.0 (6.42)	72.7 (5.25)	71.9 (5.09)	72.0 (5.55)	0.497
Median	69.0	72.0	71.0	71.0	
p_25_ ~ p_75_	67.5 ~ 76.0	70.0 ~ 76.0	69.0 ~ 75.0	68.0 ~ 76.0	
BMI, kg/m^2^					
N (Nmiss)	24 (0)	33 (0)	26 (0)	83 (0)	
Mean (STD)	24.8 (2.94)	24.0 (2.76)	24.9 (3.18)	24.5 (2.94)	0.416
Median	24.4	24.0	23.8	24.0	
p_25_ ~ p_75_	22.7 ~ 27.8	22.6 ~ 25.1	23.3 ~ 26.1	22.6 ~ 26.0	
Diabetes, *n*(%)					
No DM	21 (87.5)	25 (75.8)	22 (84.6)	68 (81.9)	0.553
DM	3 (12.5)	8 (24.2)	4 (15.4)	15 (18.1)	
GDS score					
*N* (Nmiss)	24 (0)	33 (0)	24 (2)	81 (2)	
Mean (STD)	8.3 (6.00)	9.1 (4.84)	5.4 (4.87)	7.8 (5.39)	0.029
Median	6.0	8.0	4.0	6.0	
p_25_ ~ p_75_	5.0 ~ 10.0	6.0 ~ 11.0	2.5 ~ 7.0	4.0 ~ 10.0	
Education, years					
*N* (Nmiss)	24 (0)	33 (0)	26 (0)	83 (0)	
Mean (STD)	11.8 (2.94)	13.2 (2.35)	12.9 (2.52)	12.7 (2.62)	0.119
Median	10.5	14.0	12.0	12.0	
p_25_ ~ p_75_	9.0 ~ 15.0	12.0 ~ 15.0	12.0 ~ 15.0	9.0 ~ 15.0	
AVLT-H delayed recall					
*N* (Nmiss)	24 (0)	33 (0)	26 (0)	83 (0)	
Mean (STD)	2.1 (2.12)	4.3 (2.45)	4.4 (1.60)	3.7 (2.34)	.
Median	1.5	4.0	4.0	4.0	< 0.001
p_25_ ~ p_75_	0.0 ~ 4.0	3.0 ~ 6.0	3.0 ~ 6.0	2.0 ~ 5.0	
AVLT-H recognition					
*N* (Nmiss)	24(0)	33 (0)	26 (0)	83 (0)	
Mean (STD)	18.0(3.20)	20.6 (2.38)	22.0 (1.59)	20.3(2.90)	
Median	18.0	21.0	22.0	21.0	< 0.001
p_25_ ~ p_75_	15.5 ~ 20.5	19.0 ~ 22.0	21.0 ~ 23.0	19.0 ~ 22.0	
TMT-A					
*N* (Nmiss)	24(0)	33(0)	26 (0)	83 (0)	
Mean (STD)	93.5(38.22)	70.5(24.88)	56.7 (16.77)	72.8 (30.77)	
Median	89.0	64.0	52.5	65.0	<0.001
p_25_ ~ p_75_	74.5 ~ 101.5	54.0 ~ 80.0	44.0 ~ 69.0	52.0 ~ 90.0	
TMT-B					
*N* (Nmiss)	23(1)	33(0)	26(0)	82(1)	
Mean (STD)	222.7(53.71)	182.5(57.91)	147.7(49.70)	182.7(60.96)	
Median	220.0	189.0	136.0	180.0	<0.001
p_25_ ~ p_75_	182.0 ~ 260.0	141.0 ~ 200.0	112.0 ~ 173.0	134.0 ~ 216.0	
BNT					
N (Nmiss)	24(0)	33(0)	26 (0)	83 (0)	
Mean (STD)	20.7(4.03)	23.2(3.61)	24.6 (3.02)	22.9 (3.85)	
Median	20.5	23.0	26.0	23.0	0.002
p_25_ ~ p_75_	17.5 ~ 24.0	22.0 ~ 26.0	23.0 ~ 27.0	20.0 ~ 26.0	
VFT					
*N* (Nmiss)	24(0)	33(0)	26(0)	83(0)	
Mean (STD)	16.2(5.46)	18.0(4.29)	21.1 (3.69)	18.5 (4.85)	
Median	16.0	17.0	20.5	19.0	<0.001
p_25_ ~ p_75_	12.0 ~ 20.0	14.0 ~ 21.0	19.0 ~ 24.0	15.0 ~ 21.0	
MMSE					
*N* (Nmiss)	24(0)	33(0)	26 (0)	83 (0)	
Mean (STD)	26.6(1.74)	27.0(1.95)	28.3 (1.61)	27.3 (1.90)	
Median	27.0	27.0	28.5	28.0	0.002
p_25_ ~ p_75_	25.0 ~ 28.0	26.0 ~ 28.0	27.0 ~ 30.0	26.0 ~ 29.0	
MOCA					
*N* (Nmiss)	24 (0)	33 (0)	26 (0)	83 (0)	
Mean (STD)	22.4 (3.09)	23.2 (3.06)	27.0 (2.13)	24.1 (3.39)	
Median	22.5	23.0	27.0	25.0	<0.001
p_25_ ~ p_75_	20.0 ~ 24.5	21.0 ~ 25.0	27.0 ~ 28.0	21.0 ~ 27.0	

MCI, mild cognitive impairment; SCD, subject cognitive decline; NC, normal cognition; BMI: body mass index; HIS, Hachinski Ischemic Scale; AVLT-H, Auditory Verbal Learning Test—Huashan version. TMT, Trail Making Test; BNT, Boston Naming Test; AVFT, Animal Verbal Fluency Test; MMSE, Mini-mental State Examination; MoCA, Montreal Cognitive Assessment.

## Results

**Figure 1:**
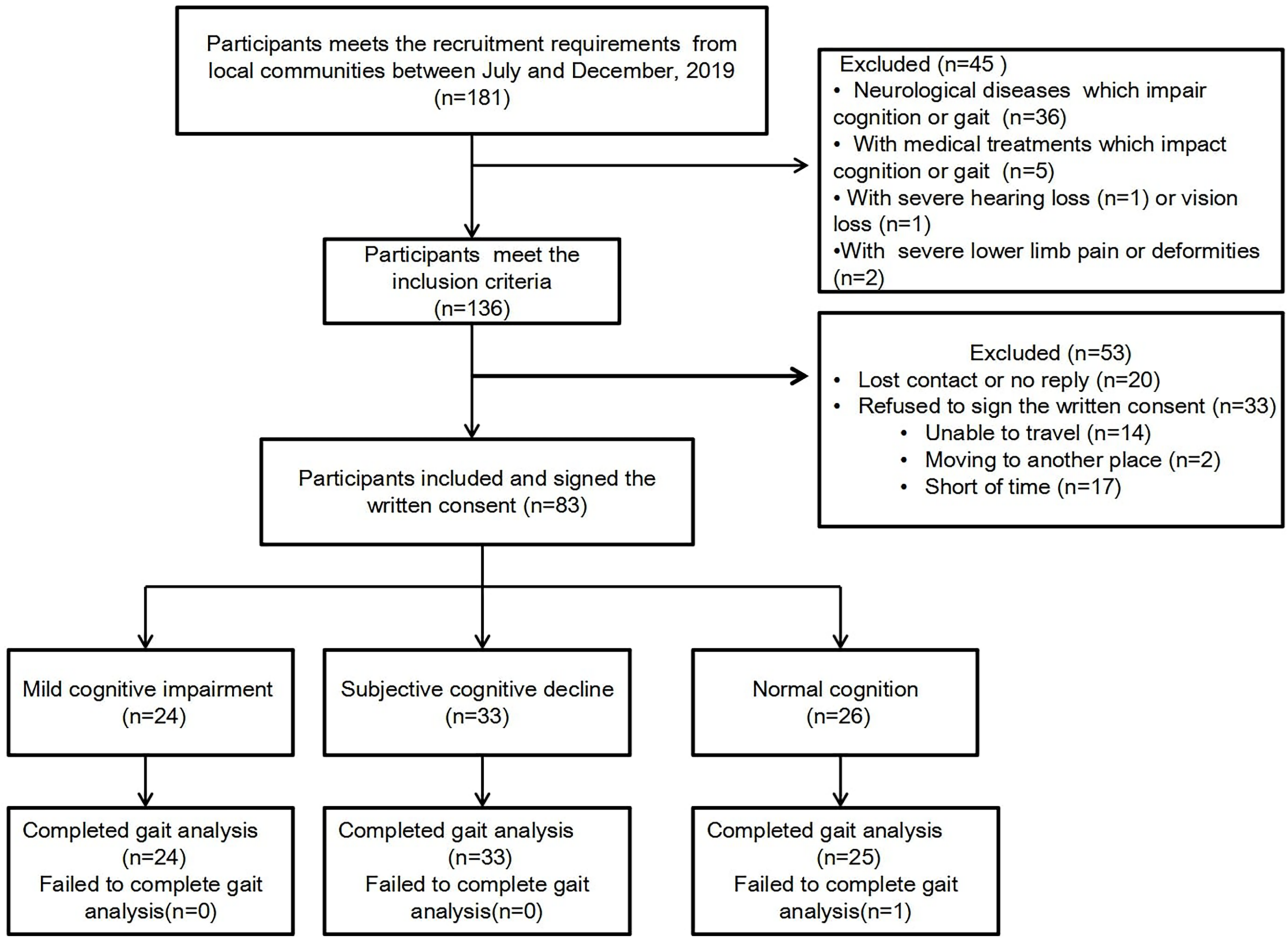
Study flow diagram.

Figure 1 shows the recruitment flow chart. At recruitment, 181 older adults were screened and 136 met the inclusion criteria. A total of 83 men and women (50% each) aged 65–83 years old initially signed up for the study. However, 53 people were excluded due to loss of contact (*n* = 20) and refused to sign the consent (*n* = 33). The reasons of not signing the consent are (1) lived too far away (*n* = 14), (2) moving to another place (*n* = 2), and (3) short of time (*n* = 17). The descriptive statistics of participants’ cognitive status and demographic characteristics are presented in Table 1. Out of 83 individuals recruited for this study, 24 were diagnosed with MCI, 33 with SCD, and 26 had normal cognition. There was no significant difference in age among the three groups, and the average age for each group was as follows: MCI 71.0 (6.42), SCD 72.7 (5.25), and NC 71.9 (5.09) (*p* = 0.497). Gender was generally balanced among the three groups, with men making up 50% of the MCI group, 45.5% of the SCD group, and 60% of the NC group (*p* = 0.112). The GDS scores were different among the groups: MCI 8.3 (6.00), SCD 9.1 (4.84), and NC 5.4 (4.87) (*p* = 0.029). The majority of the participants had 12 or more years of education. Demographic characteristics and comorbidities were balanced among the three groups. Finally, there were no significant differences with respect to age, gender, or BMI among the groups. All the cognitive assessments showed significant differences among three groups (*p* < 0.05; Table 1).

### Gait kinematic parameters of participants with MCI, SCD, and NC

We estimated the adjusted mean value for gait speed, knee peak extension angle, and DTC of different interventions, as well as the severity of cognitive disorder by the mixed-effect linear regression model and the results are shown in Table 2.

**Table 2:** The adjusted mean for speed, Knee extension angle, and DTC of different DT conditions and severity of cognitive disorder^*^.

Gait parameter	Task	MCI^a^ (*N* = 24)	SCD^a^ (*N* = 33)	NC^a^ (*N* = 25)	Total^a^ (*N* = 82)	MCI-SCD^b^	*p*	MCI-NC^b^	*p*	SCD-NC^b^	*p*
Speed (m/s)											
	DT calculation	0.865 (0.039)	0.984 (0.032)	0.968 (0.039)	0.939 (0.024)	−0.119 (−0.213,-0.026)	**0.013**	−0.103 (−0.202,-0.004)	**0.043**	0.016 (−0.08,0.113)	0.733
	DT naming animals	0.877 (0.039)	0.948 (0.033)	0.888 (0.039)	0.904 (0.024)	−0.07 (−0.165,0.025)	0.145	−0.011 (−0.112,0.09)	0.83	0.059 (−0.038,0.157)	0.23
	DT story recall	0.831 (0.039)	0.93 (0.032)	0.844 (0.039)	0.869 (0.024)	−0.099 (−0.193,-0.005)	**0.04**	−0.013 (−0.113,0.087)	0.794	0.085 (−0.011,0.182)	0.082
	DT words recall	0.897 (0.038)	0.974 (0.032)	0.945 (0.038)	0.939 (0.024)	−0.077 (−0.169,0.016)	0.103	−0.047 (−0.146,0.051)	0.338	0.029 (−0.066,0.124)	0.544
	ST	1.039 (0.044)	1.108 (0.037)	1.064 (0.044)	1.07 (0.026)	−0.069 (−0.177,0.039)	0.208	−0.025 (−0.14,0.09)	0.666	0.044 (−0.066,0.154)	0.431
	P for interaction	0.089									
	P for task	**<0.001**									
	P for cognitive status	0.1560									
Knee peak extension angle (degree)											
	DT calculation	−0.096 (1.318)	−2.939 (1.109)	−3.403 (1.319)	−2.146 (0.78)	2.843 (−0.444,6.131)	0.089	3.308 (−0.206,6.821)	0.065	0.464 (−2.881,3.809)	0.783
	DT naming animals	−0.183 (1.261)	−3.512 (1.06)	−2.976 (1.262)	−2.224 (0.751)	3.329 (0.198,6.46)	**0.037**	2.793 (−0.551,6.136)	0.1	−0.537 (−3.728,2.655)	0.738
	DT story recall	0.168 (1.24)	−1.978 (1.042)	−5.343 (1.254)	−2.384 (0.743)	2.146 (−0.925,5.217)	0.168	5.511 (2.211,8.811)	**0.001**	3.365 (0.209,6.521)	**0.037**
	DT words recall	−0.096 (1.11)	−2.425 (0.925)	−5.077 (1.11)	−2.533 (0.674)	2.33 (−0.375,5.034)	0.09	4.981 (2.097,7.866)	**0.001**	2.652 (−0.121,5.425)	0.061
	ST	−0.557 (1.121)	−3.404 (0.939)	−4.643 (1.137)	−2.868 (0.683)	2.847 (0.106,5.587)	**0.042**	4.086 (1.143,7.03)	**0.007**	1.24 (−1.596,4.076)	0.386
	P for interaction	**0.021** *									
	*P* for task	0.79									
	*P* for cognitive status	**<0.01**									
DTC											
	DT calculation	0.146 (0.027)	0.094 (0.022)	0.093 (0.027)	0.111 (0.016)	0.052 (−0.013,0.118)	0.115	0.052 (−0.017,0.122)	0.138	0 (−0.067,0.067)	0.998
	DT naming animals	0.141 (0.026)	0.129 (0.021)	0.162 (0.026)	0.144 (0.016)	0.012 (−0.051,0.074)	0.71	−0.021 (−0.087,0.046)	0.541	−0.032 (−0.096,0.032)	0.32
	DT story recall	0.188 (0.025)	0.146 (0.021)	0.204 (0.025)	0.179 (0.015)	0.042 (−0.019,0.102)	0.172	−0.016 (−0.081,0.048)	0.616	−0.058 (−0.12,0.004)	0.066
	DT words recall	0.12 (0.025)	0.102 (0.021)	0.114 (0.025)	0.112 (0.015)	0.018 (−0.043,0.079)	0.563	0.006 (−0.058,0.071)	0.845	−0.011 (−0.074,0.051)	0.718
	P for interaction	0.055									
	P for task	**<0.001**									
	P for cognitive status	0.49									

* adjusted variable: gender, age, BMI Diabetes, GDS score, Education year. DT: dual-task, ST: single task, DTC: dual-task cost.

a Variable expressed as mean (se).

b Variable expressed as mean (95% CI).

Values in bold are statistically significant, i.e., *p*-values < 0.05.

### Gait speed

The results of mixed-effect linear regression model analysis showed a significant effect (*p* < 0.001) of task-adjusted gait speed for DT story recall and was the slowest compared to all other DTs and ST in all the groups (MCI, SCD, and NC; Table 2). The adjusted gait speed under single task was faster than 1 m/s in all the three groups. Additionally, the adjusted gait speed for the MCI group was the slowest compared to the SCD and NC groups, under all DT walking conditions.

We also found a difference in gait speed under DT calculation between the MCI and NC groups [−0.103 (95%CI: −0.202, −0.004), *p* = 0.043], and between the MCI and SCD groups [−0.119 (95%CI: −0.213, −0.026), *p* = 0.013]. Although the statistical significance disappeared after adjusting for multiple comparisons, a difference in gait speed of more than 0.1 m/s can be considered clinically meaningful. Furthermore, the difference in gait speed of the DT story recall between the MCI and SCD groups was also significant (−0.099 (95%CI: −0.193, −0.005), *p* = 0.04). On the other hand, the difference in gait speed for the DT naming animals, DT words recall, and ST between the MCI, SCD, and NC groups was not significant (Table 2).

### Knee peak extension angle

**Figure 2:**
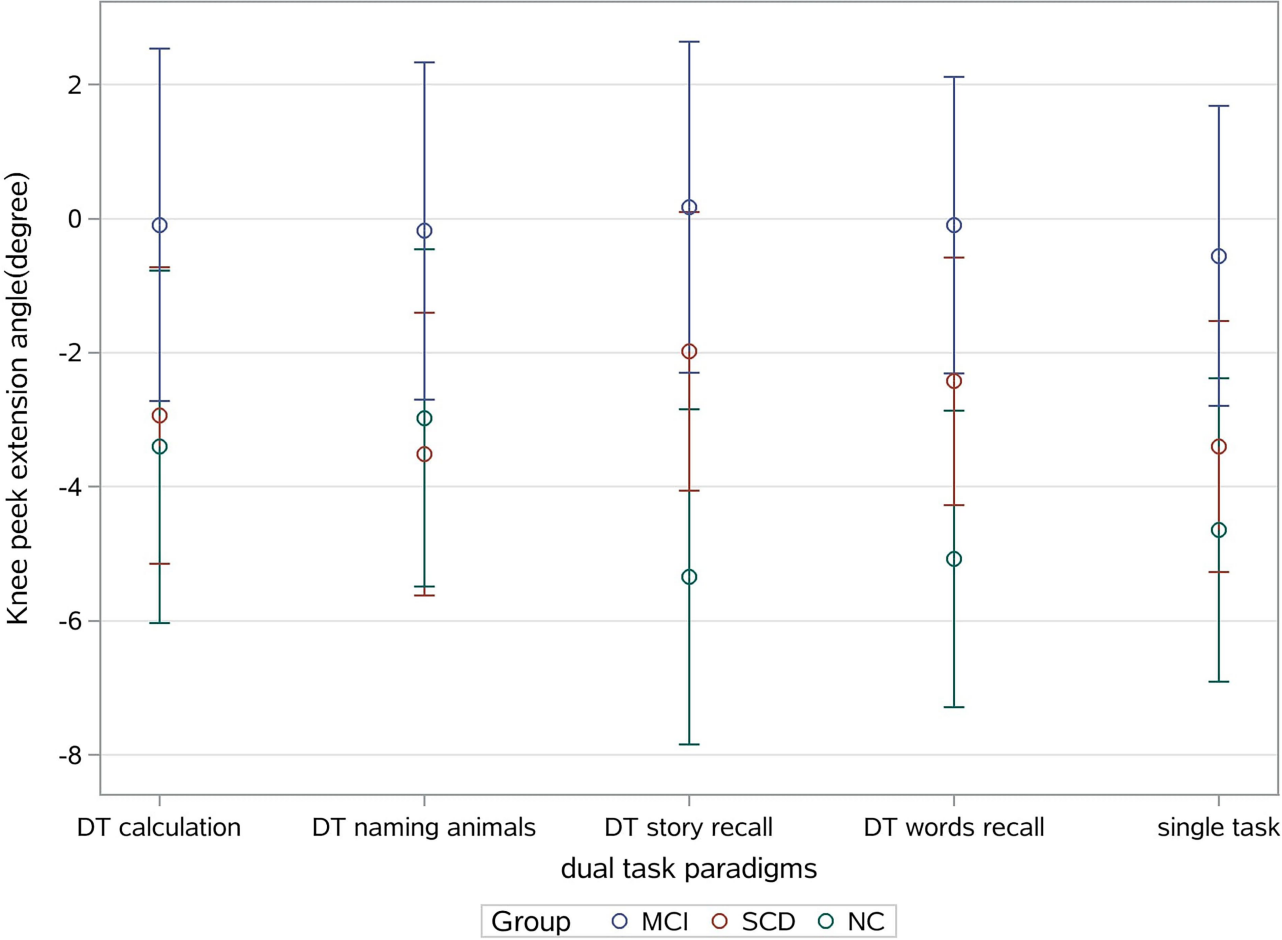
Knee peak extension angle and cognitive status of participants.

The knee peak extension angle was bigger in the MCI group compared to the SCD and NC groups under all DT and ST conditions (Table 2). We had also observed a significant difference in the knee peak extension angle under the DT story recall and DT words recall, which could distinguish MCI from NC (*p* = 0.001). Furthermore, a significant interaction effect of task and cognitive status was observed (*p* = 0.021). Figure 2 shows knee peak extension angles for the three groups under different task conditions. While there was no difference between the SCD and NC groups under the DT calculation and DT naming animals, we did observe a significant difference between the SCD and NC groups for the DT story recall and DT naming animals (3.901 (95%CI: 1.148, 6.655), *p* = 0.006), as well as a difference between the SCD and NC groups for the DT story recall and DT calculation (2.901 (95%CI: −0.302, 6.104), *p* = 0.075).

### Dual-task cost

**Figure 3:**
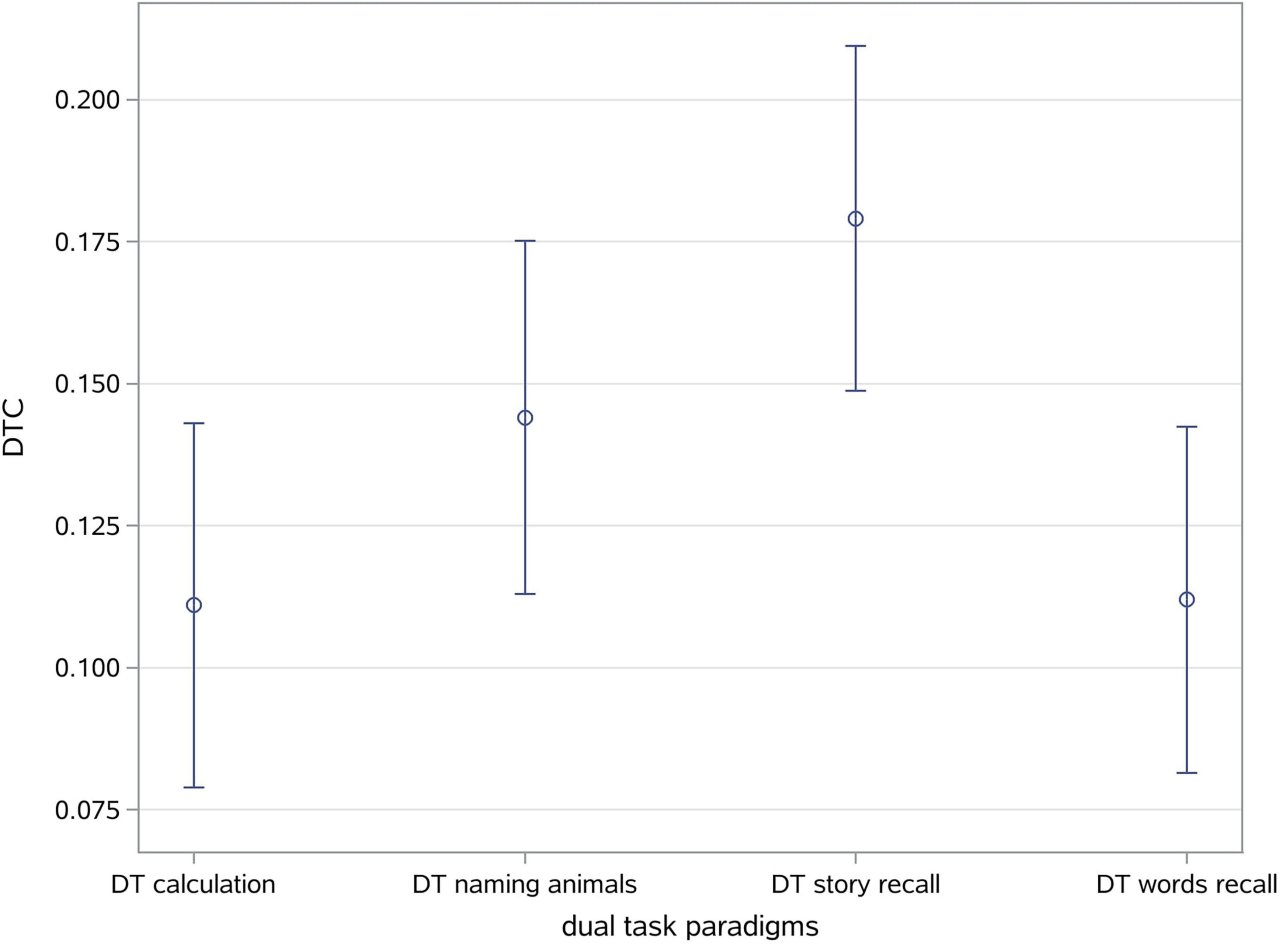
Mean difference in dual-task cost among various dual-task paradigms.

A significant effect of task on DTC was observed (*p* < 0.001) in all the groups, but no significant differences of DTC under each task were found among three groups. The difference of DTC under story recall was noticeable as −0.058 [95%CI: (−0.12, 0.004), *p* = 0.066] between SCD and NC group. Meanwhile, the DTC was higher under the DT story recall compared to DT calculation, DT naming animals, and DT words recall (Table 2). The difference in DTC between the story recall and calculation dual-tasks was 0.068 [95%CI: (0.047, 0.090), *p* < 0.001]. Furthermore, the difference in DTC between DT story recall and DT naming animals was 0.035 (95%CI: 0.019, 0.051, *p* < 0.001), and the difference between DTC for DT story recall and DT words recall was 0.067 (95%CI: 0.048, 0.086, *p* < 0.001; Figure 3).

## Discussion

In this study, we found that a novel gait parameter under DT conditions was effective in discriminating MCI patients from healthy controls. Gait kinematics, especially knee peak extension angle, was significantly bigger in MCI group compared to NC group under the DT story recall, DT words recall, and ST. We also found that DTC was significantly higher under the DT story recall compared to all other DT paradigms. The key findings in gait kinematics could be an important step forward in developing clinically validated measures for MCI-related functional deficits, and could aid in the early diagnosis of cognitive disease (Ghoraani et al., 2021).

Our results showed slower gait speed under DT condition compared to ST condition in MCI, SCD, and NC individuals, which is in line with the previous findings. Ghoraani et al. (2021), Montero-Odasso et al. (2020), and Ramírez and Gutiérrez (2021) have all shown that slowed gait speed while dual-tasking can not only differentiate MCI from NC individuals, but can also predict its progression to dementia. A recent study showed that most of the spatiotemporal gait variables could discriminate between dementia and cognitively intact individuals under single and dual tasks (Bovonsunthonchai et al., 2022). The DT in this study is counting backward which is similar to the DT calculation in our study. We found gait speed under DT calculation and DT story recall could distinguish MCI from NC group as well. Furthermore, our new finding is that gait speed under DT calculation could also distinguish MCI from SCD group, and gait speed under DT story recall could distinguish MCI from NC group. However, our results showed that the gait speed under ST, DT naming animals, and DT words recall could not distinguish different groups. Those findings indicated that different cognitive tasks have diverse interferences on walking performance, which could be affected by severity of cognitive impairment and the deficits of different cognitive domains. Further studies are needed to investigate the gait interference of memory tasks in Alzheimer’s dementia and SCD population.

Our previous work has shown that knee kinematics during level walking are significantly different in patients with MCI and NC (Zhong et al., 2021), and our new finding regarding knee peak extension angle under DT story and words recall could significantly differentiate MCI from NC group. This differences of knee joint angle are around 5°, which is clinically noticeable and meaningful. A bigger knee extension angle indicated worse knee control during standing phase, which might aggravate the walking instability and increase the falling risk of MCI patients. Reduced knee extension during stance phase was found in elderly individuals, suggesting that they favored a flexed-knee gait possibly either to give assistance in weight acceptance or to increase knee joint stability (Begg and Sparrow, 2006). However, an impairment of cognition may eliminate this age-related adaptation, leading to worse knee control during DT walking. The peak knee extension angle was found to be highly correlated with walking performance and self-reported disability in elderly with osteoarthritis (Maly et al., 2006), and its clinical significance in patients with MCI was firstly reported by our team. Therefore, functional assessments for MCI should not only include cognitive performance but also consider gait kinematics, in order to improve their functional independence in clinical interventions. Attention should also be given to strength training of knee extensors and flexors to improve knee control during ST and DT walking.

A high DTC is associated with an increased risk of progression to dementia (Montero-Odasso et al., 2017). Whether the DTC could discriminate MCI from NC remains inconsistent. Our results are in line with previous studies that DTC under DTs cannot distinguish MCI from normal elderly (Åhman et al., 2020; Jayakody et al., 2020). While others found significant differences of DTC between MCI and normal group (Zak et al., 2021; Zheng et al., 2022). The conflict of findings may due to the different inclusion criteria of MCI participants and different cognitive tasks, which may have different interferences in walking performance.

Previous studies have found that the sensitivity of DT gait assessment differs depending on the difficulty of the cognitive task. Arithmetic tasks with high cognitive demand such as calculation (such as counting by serial 3 or 7 s) and verbal fluency (naming animals) tasks are often used to elicit certain MCI-specific gait patterns. These DT paradigms could potentially discriminate MCI patients from patients with Alzheimer’s disease and healthy individuals (Åhman et al., 2020). Maintaining balance and speed while dual-tasking is a complex function that requires trunk stability, intact autonomic, and sensorimotor nervous systems. Therefore, dual-tasking requires a higher degree of balancing skills, attention, and executive function than single-tasking. As MCI is the transitional state from normal aging to Alzheimer’s disease, the use of dual-tasking with memory tasks seems ideal for observing gait changes in this population.

Cognitive decline with age is primarily observed in the domains of working memory and executive functions which results in reduced attention, postural control, and processing speed (Ramírez and Gutiérrez, 2021). However, older adults also show declines in physical functions such as loss of muscle mass, motor control, and balance (Granacher et al., 2011; Cohen et al., 2016). Therefore, age-related physical and cognitive decline are related functions that can negatively impact the quality of life and independence at older ages (Martin et al., 2011). DT walking relies on a complex neuronal network that consists of primary/supplementary motor area, hippocampus, frontal cortex, occipital cortex, and cerebellum. Although the exact mechanism of gait speed reduction is not known, it is suggested that it might be due to reduced attention resources and is in direct correlation with gray matter volume in frontal cortical regions in MCI patients (Allali et al., 2019).

The use of the DT paradigm exposes cognitive deficits through the simultaneous use of attention-demanding resources (Bahureksa et al., 2017). The story recall test is similar to an everyday memory demand that requires more attention, better learning ability, and good language comprehension of the listener (Baek et al., 2011), and may therefore provide crucial information about the coding, storage, and retrieval process of the memory system. Loss of episodic memory may further be an indicator of the early cerebral atrophy and hippocampal shrinkage that occur during the early stages of cognitive decline. Studies have shown that certain gait parameters such as slowing of gait speed in older adults are associated with reduction in memory and processing speed and therefore can provide diagnostic insights into specific cognitive domains (Chudoba and Schmitter-Edgecombe, 2020). For example, Toots et al. (2019) have found that gait speed is strongly associated with global cognition and executive functions in cognitively impaired individuals. A worse DT gait performance was found to be associated with volume reduction in the entorhinal cortex (Sakurai et al., 2019). Our previous findings have shown that gait kinematics in ST condition differ among older adults with MCI, SCD, and individuals with normal cognition (Zhong et al., 2021). In addition, our recent findings have shown that cognitive impairment can also impact DT gait kinematics in older adults. It is possible that cognition and gait share certain brain regions and control processes such as gray, white matter, and frontal brain regions and their deterioration impact on gait kinematics and kinetics. Further studies are recommended to explore the changes of DT-related brain functional network in cognitive impairment participants.

## Strength and limitations

The strength of our study is that we have used well-studied DT gait parameters and our findings are clinically relevant for the assessments of MCI patients. In addition, the changes of knee peak extension angle we observed during dual-tasking indicated a worse knee control in MCI compared to NC individuals. The gait parameter under DT story recall showed more sensitive to discriminate MCI from normal elderly. One limitation of our study is that we did not randomize the order of DT paradigms but captured the gait data in a constant order for all the participants, which may lead to some learning effect in the second or third trial of each paradigm.

Another limitation of our study is that it is a cross-sectional study and whether DT performance is related to AD progression in MCI and SCD remains unknown. Future research utilizing larger sample size with a longitudinal approach will be crucial in addressing the long-term and large-scale effects of dual-tasking on cognition in the elderly population.

## Data availability statement

The raw data supporting the conclusions of this article will be made available by the authors, without undue reservation.

## Ethics statement

The studies involving human participants were reviewed and approved by the Ethics committee of The First Affiliated Hospital of Nanjing Medical University. The patients/participants provided their written informed consent to participate in this study.

## Author contributions

YZ and TW completed the funding application, managed and coordinated the study. NA, JL, and HT applied for the ethical application, completed the statistical analysis and drafted the manuscript. WP, YT, and HW screened and diagnosed the participants, collected the data, and analyzed the characteristics of the participants. QZ, YG, HW, and CS collected the gait analysis data and completed the gait parameters analysis. MX and TW provided the research ideas, guided the study design and study process and revised the manuscript. All the authors contributed to the article and approved the submitted version.

## Funding

This work was supported by the National Key R&D Program of China (Grant Nos. 2018YFC2001600 and 2018YFC 2001603), Nanjing Municipal Science and Technology Bureau (Grant No. 2019060002), and National Natural Science Foundation of China (NSFC) (Grant Nos. 81971237).

## Conflict of interest

The authors declare that the research was conducted in the absence of any commercial or financial relationships that could be construed as a potential conflict of interest.

## Publisher’s note

All claims expressed in this article are solely those of the authors and do not necessarily represent those of their affiliated organizations, or those of the publisher, the editors and the reviewers. Any product that may be evaluated in this article, or claim that may be made by its manufacturer, is not guaranteed or endorsed by the publisher.
